# Supplementary material for: The View of the French Dog Breeders in Relation to Female Reproduction, Maternal Care and Stress during the Peripartum Period
Source: Animals (Basel). 2020 Jan 17;10(1):159. doi: 10.3390/ani10010159 (PMC7022537; doi:10.3390/ani10010159)
Supplement: Supplementary file 1 [file animals-10-00159-s001.pdf]

Parturition and Maternal Behaviour Survey

2018

About your kennel

Contact details

Your name: ….

Your firstname: ….

Name of your breeding facility: ….

Address: …. Postcode: … City: ….

Phone number:…

Email: ….

Q1 Is your kennel?

(Please tick as many boxes as apply)

- A family activity .... ☐
- A professional activity .... ☐
- Other **please specify** .... ☐

____

Q2 How many females (between 1 and 7-years old) are in the reproduction program?

(Please tick one box only)

- Less than 5 .... ☐
- 6 to 10 .... ☐
- 11 to 30 .... ☐
- 31 to 50 .... ☐
- More than 50 .... ☐

Q3 How many litters are born per year?

(Please tick one box only)

- Less than 5 .... ☐
- 6 to 10 .... ☐
- 11 to 30 .... ☐
- 31 to 50 .... ☐
- More than 50 .... ☐

Q4 Which breeds categories are represented in your kennel? (based on dog breed groups)

(Please tick all boxes that apply)

Group 1 (Sheepdogs and cattle dogs) .... ☐
Group 2 (Pinschers/schnauzers/molossoid breeds/Swiss mountain) .... ☐
Group 3 (Terriers)............ ☐
Group 4 (Dachshunds)............ ☐
Group 5 (Spitz and primitive types)............ ☐
Group 6 (Scenthounds and related breeds)............ ☐
Group 7 (Pointers and setters)............ ☐
Group 8 (Retrievers - flushing dogs - water dogs).. .... ☐
Group 9 (Companion and toy dogs)............ ☐
Group 10 (Sighthounds)............ ☐

Q5 Which breeds are represented in your kennel?

____

____

____

Q6 Where are your bitches housed most of the time? (apart from parturition period)

(Please tick all boxes that apply)

Inside the house .... ☐
In the garden .... ☐
In a kennel facility............ ☐
Other **please specify** .... ☐

____

Q7 How often are the bitches in contact with people?

(Please tick one box only)

All the time or most of the time............ ☐
Half of the working day............ ☐
Twice per day............ ☐
Once per day............ ☐
Other **please specify** .... ☐

____

Your breeding program

Q8 Do you use any technique to estimate the breeding time?

(Please tick all boxes that apply)

- Vaginal smear ☐
- Progesterone level ☐
- Measurement of electrical conductivity of vaginal mucus ☐
- Other **please specify** ☐

____

Q9 How do you estimate the whelping time?

(Please tick as many boxes as apply)

- No specific technic ☐
- I use the last day of breeding ☐
- I use the ovulation time ☐
- Body temperature ☐
- Bitch's behaviour change ☐

please specify: ______________________________

Q10 Once the bitch is confirmed pregnant what are the changes in the housing?

(Please tick one box only)

- No changes ☐
- Same housing but introduction of a whelping box ☐
- Change to the maternity area before parturition ☐
- Other **please specify** ☐

Q11 During the time in the maternity, do the bitches have access to an exercise area ?

(Please tick one box only)

- Yes ☐
- No ☐

Q12 During the time in the maternity, does the housing sytem allow the bitch to?

(Please tick one box only)

- Access outside all the time ☐
- Leave the maternity twice/day ☐
- Leave the maternity once/day ☐
- Other ☐

please specify: ______________________________

At parturtion

Q13 What is the most common time of whelping for your bitches?

(Please tick one box only)

- in the morning - from 06:00 to 12:00 ☐
- in the afternoon - from 12:00 to 18:00 ☐
- in the evening - from 18:00 to midnight ☐
- At nighttime - from midnight to 06:00 ☐

Q14 How do you monitor the whelping?

(Please tick one box only)

- I stay close to my bitch ☐
- I have a trainee staying with the bitch ☐
- Camera monitoring ☐
- Acoustic surveillance system ☐
- No observation ☐
- Other ☐

please specify: ______________________________

Maternal behaviour at whelping time

Q15 When do you consider a bitch very stressed at the time of whelping?

(Please tick as many boxes as apply)

- The bitch moves abnormally frequently ☐
- The bitch barks and/or makes other sounds ☐
- The bitch tries refuses to enter the whelping box ☐
- The bitch tries to tear off the whelping box ☐
- The bitch is aggressive towards people ☐
- The bitch is aggressive towards other dogs/bitches ☐
- Other important behaviour **please specify** ☐

____

Q16 What are the signs of a non-stressed bitch during whelping for you?

(Please tick as many boxes as apply)

- Isolating and searching for a quiet place ☐
- Looking for human contact ☐
- Sitting in the whelping box ☐
- Happy barking ☐
- Other important behaviour **please specify** ☐

Q17 Do you use products or methods to reduce the stress of the bitch around whelping?

(Please tick as many boxes as apply)

- I spend more time with the bitch ☐
- I use natural products (pheromones, Bach flowers, natural ingredients…) ☐
- I play music in the maternity ☐
- None ☐
- Others ☐

If you selected "*Others*" or "*natural products*" **please specify**: ____________

Q18 If you're using products or methods to reduce the stress of the bitch around whelping, is is?

(Please tick as many boxes as apply)

- Occasional, when a bitch is identified as stressed ☐
- Systematic, preventatively ☐

Maternal behaviour after whelping

Q19 How much time do bitches usually need to adjust to maternal role?

(Please tick one box only)

- Right after puppies' birth ☐
- 1 or 2 days after puppies' birth ☐
- One week after puppies' birth ☐
- Two to four weeks ☐
- More than a month ☐

Q20 What are the signs of a bitch stressed by motherhood?

(Please tick as many boxes as apply)

- The bitch refuses to stay with her puppies ☐
- The bitch often moves her puppies to different places ☐
- The bitch refuses to let her puppies nurse ☐
- The bitch is agressive towards the puppies (grunt and bite) ☐
- The bitch prefers to be with humans ☐
- Other **please specify** ☐

____

Q21 When a bitch is stressed after whelping, do you use products or methods to reduce her stress?

(Please tick as many boxes as apply)

I spend more time with the bitch.. ☐
I use natural products (pheromones, Bach flowers, natural ingredients…).. ☐
I play music in the maternity.. ☐
None .... ☐
Others... ☐
If you selected "*Others*" or "*natural products*" **please specify**: ____________________

Q22 If you're using products or methods to reduce the stress of the bitch after whelping, is is?

(Please tick as many boxes as apply)

Occasional, when a bitch is identified as stressed.. ☐
Systematic, preventatively.. ☐

Q23 Which of the following behaviour(s) are observed around parturition in most of your bitches?

(Please tick as many boxes as apply)

Nesting .... ☐
Eating the placenta... ☐
Licking the puppies... ☐
Walking in circles.. ☐
Transporting the puppies with the mouth to different places....................... ☐
Laying down and standing up multiple times.. ☐
Frequent barking .... ☐
Loud crying.. ☐
Other .... ☐

please specify: ____

Q24 Behaviour problems are more common?

(Please tick one box only)

At the first parturition .... ☐
At the second parturition and beyond... ☐
Not applicable, I never experienced any problem .... ☐

Q25 Do your bitches growl if someone but you approaches the nest?

(Please tick one box only)

Yes .... ☐
No... ☐

Q26 Do your bitches allow someone but you manipulate their puppies?

(Please tick one box only)

Yes ☐

No ☐

Q27 Do your bitches prefer your presence to their puppies?

(Please tick one box only)

Yes ☐

No ☐

Q28 Do you consider bitches to display a more maternal attitude?

(Please tick one box only)

At the first parturition ☐

At the second parturition and beyond ☐

No difference ☐

Q29 How often do you have problems such as a bitch refusing her puppies?

(Please tick one box only)

Never ☐

Sporadically -10 to 30% of the whelping ☐

Regurlary – 30 to 50% of the whelping ☐

Frequently – 50 to 70% of the whelping ☐

Most of the whelpings > 70% ☐

Q30 How often do you have problems such as a bitch eating one or several puppies (cannibalism)?

(Please tick one box only)

Never ☐

Sporadically -10 to 30% of the whelping ☐

Regurlary – 30 to 50% of the whelping ☐

Frequently – 50 to 70% of the whelping ☐

Most of the whelpings > 70% ☐

Q31 How often do you have problems such as a bitch with no lactation?

(Please tick one box only)

- Never ☐
- Sporadically -10 to 30% of the whelping ☐
- Regurlary – 30 to 50% of the whelping ☐
- Frequently – 50 to 70% of the whelping ☐
- Most of the whelpings > 70% ☐

Q32 In general, the bitch will stay with her puppies for?

(Please tick one box only)

- 4 weeks ☐
- 5 weeks ☐
- 6 weeks ☐
- 7 weeks ☐
- 8 weeks ☐
- ≥ 9 weeks ☐

Q33 A maternal bitch?

(Please tick as many boxes as apply)

- is friendly with her puppies all the time ☐
- is protective and does not allow to approach her puppies ☐
- licks often her puppies ☐
- nurses their puppies frequently ☐
- stays with her puppies and refuses to leave the nest ☐
- No particular clue of a "good maternal behaviour" in a bitch ☐
- Other important behaviour **please specify** ☐

____

Maternal behaviour in general

Q34 Please describe any behaviour of the bitch towards their puppies that you consider as THE MOST important

____

____

____

____

Q35 What are, for you, abnormal behaviours from a bitch towards puppies?

Q36 If you’re raising several breeds, have you notices differences in maternal behaviour according to breed? Please provide details.

Thanks again for your time and willingness to help us with these questions.
